# The Hospital Nursing Supplement

**Published:** 1893-06-03

**Authors:** 


					The Hospital, June 3, 1893 Extra Supplement
II
Zht |f?osuttal" fluvsmcj Mivvov
Being the Extra Nubbins Supplement of "The Hospital" Newspapeb.
[Contributions for this Supplement should be addressed to tho Editor, The Hospital, 428, Strand, London, W.O., and should have the word
" Nursine" plainly written in left-hand top oorner of the envelope,]
IRews from tbe iRursing TOorlD.
THE ROYAL WEDDING.
A very general desire has been expressed by the
Pension Fnnd Nurses to give their Royal Highnesses
the Duke of York and Princess May a wedding present.
This oft-repeated suggestion has been carefully con-
sidered in all its aspects, and there is a feeling that it
would be a pity that the hardly-earned money of the
nurses should be spent in giving a wedding present to
those who will have so many others. We hope, there-
fore, that the Pension Fund Nurses and others who
wished to join in such a present, will understand how
thoroughly their kind wishes and good feelings are ap-
preciated by those whom they desire to honour. It would
be a good way of giving expression to the cordial feeling
of the nurses, if arrangements could be made to take
advantage of the general holiday. The nurses might
commemorate the Royal wedding by spending the day
together, either by a river picnic or sea-trip, or in some
other way. We shall be glad to hear the opinion of
the nurses themselves as regards this suggestion,
and to do anything that may be necessary, if it be a
general wish, to organise such a celebration. This
proposal would have the advantage of giving the
nurses an enjoyable holiday, whilst it testified in a
pleasant and practical manner to the loyal sentiments
and kindly feelings which they entertain towards the
Bon of the illustrious President of the R.N.P.F. and
his future bride.
THE NURSE DOLLS.
With the view of making The Hospital Collection
as complete as possible, and also to avoid the reproduc-
tion of the same uniforms, we shall be glad if our readers
will continue to keep us informed of their proposed
contributions of dolls. It is a pity to risk repetition
when it can be avoided by sending a post-card to
the office to inquire whether special uniforms have
already been promised to the collection. We have set
our minds on having a completely representative party
?f Nurse Dolls, which will always be available for
reference. Many nurses will comprehend the practical
value of an advantage in which they will freely share,
and we are well aware of the cordial sympathy and
support which has been already evinced by every reader
interested in this particular subject. Dolls should be
received at 428, Strand, by June 17th ; they should be
addressed to the Editor and marked " Dolls " in left-
hand bottom corner of address card.
THE LATE DR. HADDEN.
The nurses belonging to that successful association,
the Nurses' Co-operation, 8, New Cavendish Street,
have lost a most valuable friend in Dr. Walter Baugh
gladden, who died on May 26th, after only a week's
Alness. Dr. Hadden was a member of the Executive
Committee, and in spite of his numerous professional
engagements, he always managed to spare time to
attend its meetings, where his practical common-sense
and his quick grasp of subjects laid before him made
bis presence exceedingly helpful. Dr. Hadden was
also Hon. Treasurer to the Co-operation, a position
involving a large expenditure of time. He was also
Honorary Physician to the nurses, many of wbom will
long and gratefully remember his kindly consideration
and indefatigable attention when they were disabled by
sickness. He was only 36, and apparently had many
years of active work before him, and his loss will be
deeply felt and regretted at St. Thomas' and at Great
Ormond Street Hospitals and by a very large circle of
friends.
MORE WORK FOR COMPETENT WOMEN.
We may indulge in reasonable hopes for the
future as regards the satisfactory sanitary pro-
gress which is under consideration. Although Dr.
Wynter Blyth's suggestions as to the increase of
staff were only partially adopted in Marylebone,
they will probably receive more adequate atten-
tion byand-by. He recommended "that the per-
manent staff be raised to eight, two of whom had
better be female inspectors." The latter would deal
with the employments and general sanitary matters
affecting members of their own sex. It would, we
imagine, be difficult to point out any line in which
competent qualified women could put their knowledge
to more practical account than in the one sketched out
by Dr. Wynter Blyth. We sincerely trust his advice
will receive the consideration it deserves. At present,
however, the Sanitary Committee have disregarded the
proposal, in so far as electing a female inspector goes
A little consideration must, however, succeed in con-
vincing most people that trained women are the only
proper " inspectors " of the personal sanitation of their
untaught sisters.
FREEDOM AND FRESH AIR.
Nurses are certainly not to be classed with other
English folks accused by tradition of "taking their
pleasures sadly." On the contrary, most nurses retain
to an enviable degree their power of enjoying rare holi-
days and unexpected treats. Few people understand
what constitutes pleasure for workers, but an anony-
mous friend of theirs has certainly accurately defined
it this spring. He (or she P) has, through a member of
the staff of the London Hospital, arranged for a succes-
sion of absolutely perfect pleasure parties. Twenty-
five of the nurses on each occasion leave Whitechapel
by an early train, and at Paddington they find a saloon
carriage, which conveys them to Taplow. Open vehicles
take them thence to a steam launch, where they embirk
for a long day to be spent in the open air, amidst
charming scenes, with a supply of excellent food,
temptingly served. There is no fatigue, nor expense,
nor anxiety on the part of the guests, which certainly
proves that the host has perfect knowledge of the " art
of resting" earnest workers. Such sympathetic com-
prehension of other people's tastes is as pleasant as the
xcii THE HOSPITAL NURSING SUPPLEMENT. June 3, 1893.
liberality which gratifies them. Many wish to give
pleasure, but few succeed so entirely as the " anonymous
friend " of the London Hospital nurses.
AN ENTHUSIASTIC MEETING.
The nurses whose promptitude and bravery on the
occasion of the fire at Kidderminster resulted in saving
twenty-seven lives have recently been presented with
watches, and the matron, Miss Mulligan, received a
timepiece. A public meeting, presided over by the
Mayor, was largely attended, and much enthusiasm
was exhibited by the parents of the rescued children.
MINISTERING CHILDREN AT LANCASTER.
Last year a sale of work, organised in connection
with the Ministering Children's League, resulted in a
sum of ?400 being handed over to the Committee of
the Lancaster Infirmary, and a cot, completely
furnished, was presented at the same time. The latter
bore the name of " The Ministering Children's Cot."
Such an excellent example deserves to be universally
followed, and we hope that the present year will
embrace a record of many more gifts equally appro-
priate and encouraging to hospital authorities.
PENNIES WELL APPLIED.
The workmen in tne Health Department of the
Corporation held a meeting at the Sunderland Town
Hall the other day, and it was decided unanimously
that each of the men would in future subscribe a penny
every week to the Sunderland Infirmary. Two of their
number were immediately chosen to make arrange-
ments for systematic collections, and the proceedings
were altogether of a most practical and satisfactory
character. The sooner such excellent plans are univer-
sally adopted in all towns, the better for the financial
position of the institutions which provide free medical
care and skilled nursing for the sick and the injured,
and the better will it also be for the independence and
self-respect of those who carry out these admirable
movements.
CONTEMPLATED CHANGES AT LEICESTER.
The proposed separation of the Private .Nursing
Institute from the District Nursing Association at
Leicester seems a wise suggestion in some respects.
The desire for the change was very generally expressed
at the annual meeting. However, there seems likely to
be a good deal of business to be settled before either
party is placed in a completely satisfactory position.
Strangely enough, whilst the District Nursing is free
from debt, and has a small balance in hand, the Private
Nurses, who are reported to be individually most
popular workers, are as an institution heavily in debt.
This is the more to be lamented, as they have had a
house rent free, that and the furniture being provided
by public subscriptions. It is, therefore, now under
discussion as to whether the private nurses in retaining
the house ought not to pay rent for it, and also to
whom does the furniture belong ? Surely the District
Nurses are still to derive some advantage from the
charitable efforts of the friends who first liberally com-
bined to start both branches of the Leicestershire
Trained Nurses' Institute.
FLEETING GLORIES!
A poem familiar to most of our readers records the
"frugal mind" possessed by John Gilpin's wife, even
when " on pleasure she was bent." But her economies
are quite eclipsed by those practised at Bradford.
Hitherto the successful candiditea in the examinations
which crown the lectures to the Infirmary probationers
have heen rewarded by gifts of books. We presume
that these were of a character to make tbem specially
acceptable to the recipients. Only nurses can fully
sympathise with the strain which studying for exami-
nations entails on probationers, and prizes are cer-
tainly well earned by such candidates. However, the
Bradford people, for some unexplained reason, have
"felt that an altogether new departure was desirable."
With the assistance of the Nurses' Institution they
have therefore purchased two medals, a gold and a
silver one. These were presented with much ceremony
the other day to the probationers who had gained the
highest number of marks at the examination. This
would be quite appropriate if they were giffs, but they
are only loans, and at the end of the year they will be
reclaimed by the Committee and bestowed, no doubt
with equal ceremony, on other successful candidates.
The honour reflected on the donors is, therefore,
practically unlimited, but the glory of the possessors is
restricted to a period of twelve calendar monthB.
SHORT ITEMS.
The Committee of the Kingston Nursing Insti-
tution, as well as the medical officers, recorded
at their annual meeting their high appreciation
of Nurse Bell's skill and untiring attention to
the patients.?Miss Winter has opened a Home for
Private Nurses at Regent Lodge, Mount Pleasant
Road, Saffron Waldon, where she can also take one or
two patients requiring massage or other special treat-
ment.?Some members of the staff of the Ci>y of
Dublin Nursing Institution presided at an attractively
stocked stall at a bazaar recently held in Dublin.?The
Perth Sick Poor Nursing Association have received
?35 16s. 3d. from an entertainment of,tableaux vivants.
?Miss Hughes, Superintendent of the Metropolitan
and National Nursing Association, will read a paper
at Chicago, entitled " The Origin and Present Work of
the Queen Victoria Jubilee Institute for Nurses."?
Award at S. Saviour's Hospital, Osnaburgh-street, is to
be devoted to the use of sick actresses, in connection
with Mrs. Willard's Hospital Bed Fund. The lease of
the hospital now belongs to the Rev. Arthur Brinckman,
and the nursing arrangement* are in the hands of the
All Saints' Sisterhood. Special accommodation is also
offered to sick nurees, and a large room on the ground
floor has been made into a kind of " rendezvous " and
reading room for nurses who can give good references
and pay a small fee.
"THE HOSPITAL" ENDOWED BED.
The liberal donation from Sister Aimee, in Africa,
was pleasantly supplemented last week by the Nurses'
collection at Eitzroy House. We think theee examples
may well be followed by all who sympathise with sick
and tired nurses. Perfect rest, amongst peaceful sur-
roundings, with freedom from responsibilities and
anxieties, will do more for a weary worker than any
other tonic. When a " run-down " nurse, whose mean"
are limited, has the expense of the compulsory holiday
constantly in her thoughts, recovery of her normal
strength is grievously hindered. A mind at ease is
needed for perfect convalescence. Surely, therefore, all
who realise this will be glad to think that their contri-
butions to " The Hospital Endowed Bed " will secure
to a succession of nurses during the current year the
change so urgently required by some of them. We
have the pleasure of acknowledging 10s. received this
week from Miss Horne, and ?3 lis. from Mr. Thoma6
Neville, makiDg a total of ?30 10s., and completing the
sum needed to support the free Bed for another year.
This is a special donation from Mr. Neville, who has
already kindly subscribed to the fund.
June 3,1893. THE HOSPITAL NURSING SUPPLEMENT. xciii
Sanitation for IRurses.
By a Sanitary Inspector.
VIII.?VACCINATION IN VARIOUS COUNTRIES.
The last Vaccination Act for this country bears the date of
1874, but as early as 1853 vaccination was made compulsory
in this country, and ten years later in Scotland, and in both
caBes has remained so ever since.
The Board of Guardians has the control over this depart-
ment, whilst each separate district under the Board has a
public vaccinator, who is required to be not only a duly-
qualified medical practitioner, but must also have received
special instruction in vaccination. Under him is an officer
whose duty it is to look up parents who neglect to have their
children duly vaccinated before the age of three months. In
Scotland the age is, however, a little later, viz., six months.
In case of neglect to comply, within the prescribed limit of
age, a special notice is sent to the parents, and if they persist
in their negligenoe they are then summoned before the magis-
trate, and fined.
There is no possible excuse for neglect, nor are the parents
ever given the chance of pleading ignorance, for at the time
of registering the child's birth a printed warning is given
to the parents that vaccination is compulsory within three
months.
There are still many people to be found who are foolish enough
to resist this most wise safeguard against a very terrible dis-
ease, and who will advance most paltry arguments in support of
their opposition. Few people have better opportunities of
enlightening the people on this most important matter than
have nurses?especially those engaged in district work?and
we hope they will take advantage of every opening they may
have to dispel such foolish and ignorant opposition. Perhaps
also the recent experiences in Leicester, which has so long
prided itself in its anti-vaccination movement, will further
help to dispel the opposition.
If any proof were needed of the use of vaccination as a
protection against small-pox, at least in its dangerous and
fatal form, the following statietics which have been care-
fully compiled by Dr. Wynter Blythe, the Medical Officer of
Health for Marylebone, and published in his Manual, should
carry conviction to the most sceptical mind. Moreover,
with the complete and efficiently carried out system of noti-
fication, as well as of registration of deaths, now in force in
this country, the accuracy of such statistics can be thoroughly
relied on :?
Vaccinated.
Percentage mortality of all ages   4*8
Percentage mortality of children under 10
years of age  1*7
Percentage mortality of persons 10 years of
age and upwards ...   5"1
Non-Vaccinated.
Percentage mortality of all ageB ... ... 49'6
Percentage mortality of children under 10
years of age  43*8
Percentage mortality of persons 10 years of
age and upwards ..  54
As regards foreign countries, we find vaccination com-
pulsory in Germany, Sweden, and Finland; whilst in
Austria, Belgium, and France, though not made actually
compulsory by law, it is so practically. It is encouraged in
every possible way, both directly and indirectly, by the re-
spective Governments of these countries. A certificate is
required of vaccination, or of due re-vaccination, from all
those who seek admission into public schools, and some other
public institutions. In some of the countries it is part of
the duty of the clergy in country distriots to impress on the
people the advantages of vaccination, and even to keep a
register of births and of unvaccinated persons in the parish,
and send it to the authorities. In Austria anyone who enter?
even as a candidate for a scholarship at a school or college
must present a certificate of vacciuation or re-vaccination.
In Belgium a similar rule prevails for all those who seek
relief for themselves or their children. Moreover, the clergy-
have to see that all children attending their " first com-
munion " have been vaccinated duly. In France so great is
the zeal of the authorities, that the poor are given a small
pecuniary reward for bringing their children to be vaccinated
or for undergoing the operation themselves. In Sweden no
child from an orphanage or asylum may be given up without
a certificate of vaccination, and in Finland such an assurance
is required of all prisoners, and persons confined in reforma-
tories, as well as of every sailor.
It is noteworthy that although in some respects Finland
is somewhab behind other countries in sanitary science, that
vaccination has been in vogue there, long before it Was so
elsewhere, viz., in 1804, whilst the State made a special
grant towards it in 1824, and[it was finally made compulsory
in 1885.
In Sweden vaccination was made compulsory in the same
year as in this country?viz., in 1853?whilst Germany only
followed in 1874. In every European country the operation
is performed gratuitously, and in most of them calf-lymph is
used in preference to human vaccine. In Stockholm the
lymph may not be used until after the calf has been killed
and proved perfectly free from disease, and in all countries
the lymph is carefully prepared at the Government depot at
the capital, under the direct supervision of the chief, and is
distributed gratis to all country districts which apply for it.
In Germany some detailed regulations are laid down, both
as regards the lancets used for vaccination, which are to be
carefully washed and disinfected in carbolic after each opera-
tion and " dried with carbolic or salicylic cotton, not ordinary
linen," and also respecting the children brought to be vaccin-
ated. They are to be " clean and properly clothed," and,
if not, they are to be sent back. We wonder whether the
German Government also provides free soap and water and
" proper clothing " for such children. Otherwise, in the case
of very poor parents, we do not see how, under ?uch condi-
tions, they can be compelled to bring up their children for
the operation, even if free ; and there might, moreover, well
be considerable difference of opinion on the part of parents
and offioials as to what constitutes " proper " clothing.
The age within which children must be vaccinated varieB
greatly in different countries ; thus, whilst in Germany it
must be performed within the first year, in many other
countries, such as Sweden and Finland, the limit of age is
two years. The remarkable difference between the limit of
age in England, which is three months, and that in two other
countries, which equal or even exceed us in their zeal for vac-
cination, such as Sweden and Finland, which fix their limit
at two years, seems to require some explanation, since if the
later age will do as well as the very early one we have
adopted, it seems a far wiser plan to postpone the operation
till one or two years of age. On the other hand, according
to Dr. Wynter Blythe'a statistics, the percentage mortality
amongst the unvaccinated is higher after ten years of a*?e
than it is before, and on this perhaps is based the postpone-
ment of age in foreign countries.
Those who are authorised to perform vaccination in various
countries are qualified medical practitioners in this country
and also in Germany. In Sweden, midwives and sacristans, and
anyone elsewho can produce proof of competent knowledge of
the course and attendant symptoms, and of the means of dis-
tinguishing it from other diseases which it resembles, are
allowed to vaccinate. All would-be vaccinators must also
show that they are able to keep the registers of vaccination
properly. In Hclsingfors, the Finnish capital, it is incum-
bent on the head of the Government depot to teach anyone,
male or female, who wishes to learn how to vaccinate, and he
has to give a certificate of competence.
m 11
xciv 7HE HOSPITAL NURSING SUPPLEMENT. jUNE 3,1893.
Ibospttal flursing tn tbe IRortb of
3n&ta.
(Br Our Indian Correspondent.)
A VISIT TO A CANTONMENT HOSPITAL.
I was jolting along from aide to side the other day in a car-
riage drawn by a couple of scraggy, ungroomed bazaar tats,
when'I was startled by a huge charger, riderless and stirrup -
less, galloping madly past me, rejoicing in his newly found
liberty.
A little further on a group of natives was congregated
round something on [the ground. Thinking I might be of
use, I stopped my ghari, and went to see what was up. The
groom was lying insensible with a big scalp wound bleeding
profusely, and a cut on his arm.
The natives seemed considerably surprised that'an English
mem sahib should deign to be interested in the accident of a
lowly Hindu. I had my ghari brought up, and into that
most uncomfortable and unadaptable conveyance put my
patient.
Finding that he was an officer's servant, I ordered the
ghari-wala (driver) to drive to either the Followers' or the
Cantonment Hospital, whichever was nearest.
Followers' hospitals are for the reception of severe cases of
sickness or accident among the Army Hospital Native Corps
and the native establishment attached to British troops.
Cantonment hospitals also are for the treatment of natives,
and are often called bazaar hospitals, being situated in close
proximity to sudder or regimental bazaars. They are always
under the superintendence of an officer of the Army Medical
Staff or of the Indian Medical Staff, it being necessary for
those who hold the appointment to have passed a certain
standard in the Hindustani language, and they have addi-
tional pay for their services. The superintendence of the
Followers' Hospital is usually attached to the staff surgeon's
billet.
My charioteer, the ghari-wala, decided in favour of the
Cantonment Hospital. Being a total stranger, I was at his
mercy, a crowd of natives manned the top of my bathing-
machine-like conveyance, the patient and myself being inside.
I thought of all I had learnt and read about first aid to the
injured, in the dim long ago, but, alas, those suggestions did
not embrace caste. I, like all novices in the country, live in
terror of breaking caste. Already I have been threatened
with an exorbitant fine for throwing dirty water over a
punkah coolie.
By the way, I have got now a splendid dodge for keeping
my punkah coolie from going to sleep. I have a fox terrier
who sleeps on my bed at night?he, like his mistress, loves
the punkah. When the coolie goes io sleep and the punkah
stops, my dog wakes up and barks at the man till he goes on
again.
But I must go on with my story. I dared not take my
pocket handkerchief to staunch the man's wound, I had
visions of paying fifty rupees to restore the man's caste.
Even charity has its limits, and there were unfortunately few
resources among his own garments?a rag of a coloured waist-
coat and a loin cloth. Each jolt and swing of the ghari
brought us in collision or sent his head against the unbend-
ing wooden Bides of the carriage. I could not pillow his head
on my shoulder, natives' heads are never nice; however, one
of the natives outside providentially popped the sais'
puggari in. I tore off the cleanest portion to stop the
bleeding, wrapt the other into a nest, placed it in the angle
of the carriage, and held his head against it, as best I could,
for a mile and a-half, till the hospital was reached, and very
bad the poor man looked when we got him there.
The sound of wheels brought out a swarm of natives, who
obsequiously made way for the native hospital assistant?
oily and sleek, with a green plush cap on hia long black locks,
a snowy white mull shirt, and a black velvet waistcoat.
Quite the smart gay Baboo.
Admittance or attendance for a time seemed doubtful.
Had I an order or a letter ? I got cross at the delay, scolded,
threatened to go to the medical officer, told him the man was
an officer's servant, gave them my card, and offered to take
all responsibility. After a lot of neediesa palaver, a charpoy
(bed) was brought and placed outside, the man lifted out and
laid on it, and then operations commenced.
A compounder arrived, who examined the wounds; the
water-carrier stood in attendance, a towel over his arm, basin
of clean water and Boap-dish in hand?these, by the way,
were not used, but only formed part of the show ; a mehber,
or sweeper, a low caste man, stood by to remove the dirty
water or lotions. Another native brought the dressings in a
wooden tray-box, containing ointments, strapping, lint,
instrument pocket-case, &c. Another native brought two
lotion bottles; tbe hospital assistant was most careful to
inquire which was the "antiseptic lotion and which was the
carbolic ? " I was considerably impressed, though I had secret
doubts as to the antiseptic properties of any of the adjunots
for dressing and ministering to the wounded. By thia time
the crowd round the patient was great, the gapers who had
arrived on my ghari were there, all the convalescent patients,
ihe numerous attendants, and last, but not least, the ghari
in close proximity, and the two poor old ponies standing in
stolid indifference.
The hospital assistant then commenced operations before
the awestruck crowd in a skilful manner. He cut off the
hair and probed unmercifully for some time, the patient
writhing and groaning. No pity, only vivid interest, and
even pleasure were depicted on the surrounding faces. At
last the work, after elaborate explanations and comments
addressed individually to myself and collectively to the
spectators, waa done, and the patient was left in peace on
the bed.
There was then nothing exciting left; one of the spectators
thrust a pair of broken stirrups in my hand, murmured
" Hawbe, Sahib's," and fled. Another gave me a fitting
embroidered cap, which, I concluded, belonged to the patient,
and I stuck it beside him on the charpoy. The most perfect
of capeline bandages already enveloped his head.
I then asked for permission to visit the hospital. Every-
thing was in excellent order, I must say. The bedding, of
course, was poor, and the natives are not used to it. They
at home sleep either on the floor on a durrie or on a charpoy,
with a little hard pillow, and a blanket flung round them, or
nothing at all, and no mattresses.
(To be continued.)
fair ant) ITlnfatr lEypenses.
Much interest has been aroused on the subject of nurses'
earnings. Until quite recently many of those engaged in
private work received but a small proportion of the fees paid
by their patients, which was an obvious injustice. Various
causes have combined to change this condition of things and
fairer wages are the outcome. However, there exists a
reverse side to the pretty modern picture of 41 Nurses receiv-
ing their own earnings." The greater freedom they now
enjoy seems to have damaged the consciences of some of
them. Frequently do we hear of exorbitant charges taking
the place of just demands. Because patients are able to pay,
a nurse has no moral right to charge them more than her
ordinary fees. When once a woman condescends to such a
practice, it will not be long ere she degrades her profession
by others of the same description. Washing-money is made
an elastic item, and so are caba. If a nurse is content to use
the convenient 'bus and the ordinary third-class carriage,
when her travelling expenses are defrayed out of her own
pocket, surely it is neither honourable nor fair to do other-
wise when employers have to pay for her journey. Small
meannesses are of daily occurance, but when they are per-
petrated by those whose standard should be exceptionally
high, it proves the acquisition of higher remuneration to
have fostered in some quarter a spirit of greed moat unworthy
of any member of the nursing profes8ion.
June 3, 1893. THE HOSPITAL NURSING SUPPLEMENT. xcv
"Keaistratton of IRurses.
THE NATURE OF THE OPPOSITION.
The following correspondence, which has appeared in the
Times, will no donbb be read with interest. It will be re-
membered that Sir Horace Davey, with the fnll concurrence
of Sir Richard Webster, impressed upon the Privy Council
that the opposition was not directed to the granting of &
charter, per se, to the Royal British Nurses' Association,
but that it was held to be objectionable to set up a chartered
register of trained nurses under the control of this Associa-
tion. The verbatim report of the proceedings before the
Privy Council, entitled " The Battle of the Nurses," pub-
lished by the Scientific Press, 428, Strand, shows that this
was so. On page 137 Sir Horace Davey states : ?
" ' My learned friend is not instructed to say that the other
objects and purposes of thiB Association are not such as
deserve approbation and commendation on the part of the
Ciown, and such aB will reasonably entitle the Association
to crave the status of a corporate body.'
"Sir Richard Webster: 'That is perfectly right. We
did not address any argument upon that point.' "
This extract clearly proves the grounds of the opposition to
have been those we have stated. Everyone may therefore con-
gratulate the Princess Christian upon having secured, a
charter for the unopposed purposes of the Association, pro-
viding no power haB been given under the Charter to set up
a chartered register. It will be seen from the following
correspondence, that it is contended that the Charter of the
Royal British Nurses' Association will not confer upon the
Association the power to establish a chartered register. The
correctness of this contention is not denied by the Hon. Sec,
of the R.B.N.A. A charter will be granted, but what sort
cf a charter ? That is the whole question.
[Correspondence in "The Times."
Canon Troutbeck, Times, May 26th, 1893 :
Sir,?I have good reason to believe that the " Register of
Nurses," originally contemplated in the charter "as drafted
on the part of the applicants," against the maintenance of
which Sir R. Webster, " according to his instructions,"
directed his main argument, baa not found place in the draft
as finally approved and authorised.
Will some one with acourate knowledge be good enough to
say in plain words, without ambiguity, whether this ia eo or
not?
Those who, on public grounds, have felt it to be their duty
to oppose the application for the charter as it originally stood
are anxious to get at the faots in this matter, now that) the
grant of a charter has been announced by the President of the
Association. I remain, Sir, your obedient servant,
J. Troutbeck, D.D., Hon. Treasurer of the Westminster
Training School for Nurses.
4, Dean's Yard, S.W., May 25th.
Dr. Bezly ThoRNE, Times, May 27 th, 1893:
Sir,?The anxiety to get at the facts, in the matter of the
charter about to be granted to the Royal British Nurses'
Association, which has impelled the Rev. Dr. Troutbeck to
ask a specific question in your issue of this day will, I feel
persuaded, not prevent his recognising that, even were it
otherwise in a position to do so, the Association could not
with propriety engage in the discussion of the details of a
document whioh has not aB yet been officially made public.?
i our obedient servant,
W. Bezly Tiiorne, M.D., Hon. Sec. R.B.N.A.
May 26th.
Mr. Burdett and Canon Troutbeck, Times, May 30fcb,
1893 :
Sir,?As one unconnected with the aotive management of
any nurse-training sohool, who has followed the proceedings
before the Privy Council throughout with some care, may I
point out that tho opposition to the application of the Royal
British Nurses' Association was directed aolely to the ques-
tion of authorising a chartered register of trained nurses, and
not to the mere incorporation of the Association.
The question asked by Canon Troutbeck goes, therefore,
to the root of the whole matter, and until that question is
answered it is very desirable that people should reserve their
judgment on the points at issue. Dr. Bezly Thome's reply
to Canon Troutbeck is a little disingenuous, having regard
to the fact that a clause alleged to have been added to the
charter was quoted in your leading article. It is noticeable
that Princess Christian's address appears to have contained
no notice of a chartered register.
In these circumstances, is it too much to request the.
authorities of the British Nurses' Association to enlighten
the public on this crucial point ??I am, Sir, &c.,
Henry C. Burdett.
The Lodge, Porchester Square, W., May 27th.
Sir,?Will you allow me to thank Dr. Bezly Thome for
his letter ?
I fully recognise and appreciate his discretion.
So far a9 there has been any premature discussion of the
points of the forthcoming charter, it is not by me that it haa
been desired, begun, or carried on. All I have done is to ask
a simple question on one specific point, of whioh much has
already been made.
I fear my question has proved inconvenient.?I remain*
Sir, your obedient servant, J. Troutbeck, D.D,
May 27th.
prince Hlfrefc ibospital, 5\>&ne?.
THE NEW NURSES' HOME.
The beautiful Nurses' Home which was recently opened at
Sydney by Lady Jersey, in the presence of a large number of
guests, is connected by a covered way with the fine Prince
Alfred Hospital. The new building is in the picturesque
style of architecture known as Queen Anne's, and it is
situated in a charming part of the celebrated hospital
grounds. It commands an extensive view over University
Park and the hospital gardens. Both externally and
internally this Australian Nurses' Home is certainly one to
be admired and imitated in both hemispheres.
When once the necessity for providing suitable quarters
for the hospital staff was acknowledged in Sydney, several
leading citizens lost no time in offering handsome donations
to form the nucleus of a fund, which rapidly increased to
over ?10,000. There was, consequently, no difficulty
experienced in arranging for the erection of a thoroughly good
and modern nurses' home. The plans were admirably
designed by Mr. H. C. Pitt, and carried out under his super-
vision by Messrs. Stuart Bros. The building consists of
three floors and a basement; the latter, through a fall in
the land, is clear of the ground, and in it are situated the
airy, well-lighted kitchens, sculleries, and pantries.
The ventilation of this department is perfect, and the whol?
floor Is kept cool and fresh. This excellent management adds
greatly to the attractions of a special kitchen which, also
situated in the basement, is devoted to the sole use of the
nurses. In it they receive courses of instruction in the
preparation and cooking of invalid dishes. This admirable
scheme deserves to be widely known, and cannot be too
highly recommended. The managers of the Sydney Hcspital
are to be congratulated, not only on having given a completely
furnished kitchen for tbis model "school of cookery," bub
also on so wisely planning to secure a temperature in it which
Bhall attract, and not repel, the attendance of pupils. In fact,,
the chief drawback of all kitchens would be removed if the
lesson taught in Australia were intelligently applied by
English architects. The tendency, at any rate in private
houses, being to give far too little consideration to this,
apartment, yet certainly the ventilation of no portion of any
dwelling is more important than that of rooms where the food
of the establishment is kept and prepared.
The fine entrance hall on the ground floor contains the
buBt of Florence Nightingale, which she eent as a gift
to the nurses. The plans of the Home were submitted
to MIbi Nightingale previons to ita erection, and she made
xcvi THE HOSPITAL NURSING SUPPLEMENT. June 3, 1893.
a most careful inspection of them, giving advice which was
valuable as coming from one whose experience of the needs
of nurses is as great as her sanitary knowledge.
A fine marble bust of Sir Alfred Roberts, unveiled by
Lady Jersey during the opening ceremony, is also placed in
the handsome hall. Cordial acknowledgment of the
services rendered to this and other Australian hospitals by
Sir Alfred Roberts must always be gratefully recorded. He
has devoted talentB, time, and money to forward the best
interests of the nursing profession, and it is fitting that all
should appreciate his good offices. These have been ably
seconded in Sydney by Sir Alfred Stephen, Sir William
Manning, the Hon. Edward Knox, Mr. Walter Lamb, Mr.
'Grafton Rosa, the late Mr. J. Fairfax, Professor Anderson
Stuarc, Dr. Norton Manning, the late Messrs. H. Allan,
Robert Hunt, and Christopher Rolleston, the Hon. T. B.
Watt, and many others.
On the same floor as the hall, which is entered by stained
glass doors, there iB the main dining hall, 42 feet by 29
feet, furnished with half-a-dozen tables, and able to accomo-
date about 50 nurses ; a strangers' waiting-room ; the nurses'
sitting-room, which is charmingly furnished, and very large
and airy; also the Matron's office, and the Matron's and
Sisters' dining-room. A lift in the scullery conveys all the
meals direct from the kitchen to the dining hall.
The very pretty pictures which adorn the walls of
the sitting-room were painted by Australian ladies, and
bought by a few friends of the hospital as gifts to the Nurses'
Home. This sitting-room and the Sisters' dining-room are
carpeted, and the floor of the Matron's office is covered with
linoleum. The latter is also laid down in corridors and on
the stairs, leaving about a foot on each side of uncovered
polished wood.
The staircases, doors, and skirting boards are all of well
polished cedar wood, and on the ground floor, near the dining-
room, there is a convenient dreseing-room for the nurses. This
pretty room is fitted with a long marble washstand, containing
six basins, supplied with hot and cold water, two large look,
ing glasses, pin trays, <fcc., and it is intended that the nurses
should go there instead of to their bed-rooms before meals.
Certainly there seems provision for every necessity in this
admirably-planned establishment. All the sanitary arrange-
ments appear to be excellent, and each bath-room contains
the facilities for a " shower " as well as an abundant supply
of hot and cold water.
On the first floor there is a delightful library furnished with
desks, &c., and a famous supply of standard works of all kinds,
besides valuable medical and nursing books. A recent donation
of ?40 worth of volumes has been received and welcomed.
Near the library is situated the nurses' work-room, which is
frequented by those for whom needlework and conversation
offer special attractions. This preserves the library as a
?quiet place for study, and permits both kinds of workers to
follow their inclinations without mutual hindrances.
The sick-room, a very cheerful apartment, is furnished
with two beds, and is prettily fitted up.
The second and third floors are occupied by the nurses'
bed-rooms, each of which contains a bedstead, spring
mattress, wardrobe with looking-glass, marble washBtand,
chest of drawers and dressing table combined, and a
triangular slab writing table, &c. There are thirty-nine of
these single roomB, and also five double rooms, which can be
shared by two Sisters or friends.
Probationers go through a course of thr^e years' training,
and have to pass three examinations before gaining their
certificates. The applications for training are far in excess
of the vacancies, and the Prince Alfred Training School for
Nurses is certain to maintain and increase its high reputa-
tion in the hands of its present Matron.
The administrative abilities of Miss McGahey are as re-
markable as her intellectual ability, and her nursing Btaff
?will rank second to none. Her gifts for organisation were
recognised and appreciated at the great London Hospital,
where, as a highly-valued sister, she did much responsible
work. Many English nurses congratulate themselves on
their acquaintance with the lady who now holds so re-
sponsible a position as that of Matron at the beautiful hos-
pital at Sydney named after Prince Alfred.
Miss McGahey at present combines the duties of Home
Sister with that of Superintendent of Nurses, but with the
increase in numbers and the accumulated responsibilities of
the extensive new home, the Committee will probably wish
at no distant date to lighten the labours of so valued a Matron.
Jfotr IReabing to tbe Sick.
MUSIC.
In reading the book of the Psalms, we are struck with the
variety which appears among them. They sing of the
variableness of God's dealings with many, they are in many
metres, nor are all tuned to one string. We find songs both
of mercy and judgment; they fit the needs of all, hence
they have been called the Christian's prayer-book and
hymnal. We may compare our hearts to musioal instruments
in the Almighty hands, which He has made it our duty to
keep in tune. If one string be too tight or too slack, there
will be no harmony to His ear. We may answer pleasantly
when the Great Musician strikes a chord of joy and gladness,
but how is it when He touches one of sorrow and humiliation
and suffering? We shiver and throb at the vibration, for
we are out of tune with our Maker; His performance does
not suit our tastes ; perhaps a string snapB, then we are
broken instruments ; but, any way, we make no melody unto
God.
A well-tuned heart should have all its strings?that is,
all its affections ready to answer every touch of God's finger,
like "David's harp of sweetest sound." If it were bo, we
should find everything that comes from His hand beautiful
in the course of time, for the sweetest harmony is produced
from some discords. " When hath a gracious heart the
soundest joys, but when it hath the deepest sorrows?
When hath it the humblest meltings, but when it hath the
most ravishing joys ? " The prophet Habakkuk, in his song,
exclaims, "Though the crop should fail and sorrow and
sickness be my portion, yet will I rejoice In the Lord, I will
j >y in the God of my salvation." This is the strain which we
should try to learn on a sick bed, and though we may find it
difficult to perfect, the labour will not be In vain, for we shall
be ready to respond harmoniously to the finger of God what-
ever strings He may touch. We should answer to the " terri-
ble things of His righteousness " with hope, fear, reverence,
humility, and repentance as quickly as when joy, delight,
love, and thankfulness are the theme.
Our loving Father would have His children wholly wrapped
up in the music he plays. He delights in seeing them submit
their tastes to Him, to be content to lose themselves in the
infinite unsearchableness of His goodness and power.^ When
we are perfected here below, we shall be ready to join in the
ravishing strains of Heaven, where the redeemed sing the
new song of Moses and the Lamb. There we shall find that
perfect chord which now and again has thrilled our very souIb
with unutterable bliss on earth, but which can only be
securely ours when we have mounted the " golden staira "
and fallen down in worship before the " Great White
Throne."
appointments.
[It is requested that successful candidates will send a copy of their
applications and testimonials, with date of election, to Thh Editor.
The Lodge, Porchester Square, W.]
Dundee Royal Infirmary?Misa M. A. Strong has been
made Matron of the Dundee Royal Infirmary. She waa
trained at the East London Children'a Hospital, and was
afterwards Charge Nurse at the Royal Infirmary, Truro;
Sister-in Charge of the Children's Hospital, Bolton; and
Night Superintendent at the London Fever Hospital. We
wish Misa Strong every auccest! in her present appointment.
TOlants ant? MorRcrs.
Will anybody kindly jfive mo the address of Miss Dally, lately a
nurse in the Samaritan Hospital, Portman Square, London ??Nurse 0.
June 3, 1893. THE HOSPITAL NURSING SUPPLEMENT. xcvii
laminations for probationers.
By a Victim.
EAKLY'efforts made to improve the theoretical knowledge of
probationers were on a much simpler scale than the present
elaborate Bystem of lectures and examinations. Naturally
the first institution of a course of regular training seemed
such a marvellous improvement on the old condition of things
that only very elementary lectures were considered needful.
"Lectures on Nursing " is the vaguest of terms now-a days,
for it may denote teaching which can be assimilated only by
fully-trained women, or it may mean merely little friendly
chats on nursing in village homes.
But the courses usually given to modern^robationers treat
of more than simple nursing detailB. They include elemen-
tary physiology and anatomy, gynaecology, &c. Looking
at the long list of questions to which intelligent answers are
required, the outsider may well feel thankful that she her-
self is not in the ranks of probationers.
Knowledge truly is power, but discretion is certainly
needed in imparting it. The quantity prescribed should
always be of digestible quality.
To the question," When should probationers be instructed ?"
we should answer, "At all times, in practical points."
Whenever difficulties arise, information should be asked and
given.
So much is learnt by intelligent observation of other
people's actions, that a looker-on is hardly aware of the
teaching which is insensibly achieved. In fact, one of the
commonest complaints of ill-balanced probationers is the
lack of adequate instruction given in the nursing depart-
ment. Generally speaking, this may be taken to show
either indolence or density on the grumbler's part, for she
has evidently wasted many valuable opportunities. Educa-
tion is carried on incessantly in the ward, and those who
profit most, probably feel the mental strain least.
It is, however, chiefly with the lectures and examinations
of probationers, that~we are concerning ourselves at present.
The lectures certainly ought not to absorb any " off duty "
time.
In every institution where this is the case, they are at once
justly considered as a kind of imposition. There is a feeling of
an injustice done with which we cordially sympathise, even
though we disapprove of the inevitable shirking of duty
which is its outcome. Recreation times should be altered,
but never curtailed, to ensure regularity, although attendance
at lectures should always, as an important part of a nurse's
training, be considered compulsory. Some institutions have
a curious custom of preserving the best lectures for the
delectation of paying probationers only, and making simple
classes of instruction suffice for the rest of the staff, but this
obviously impolitic.
A better plan ia to admit all to the lectures, but have two
?tandards of subsequent examinations, the higher for those
?urses who have originally enjoyed the advantages of a
thorough education, and the modified one for those whose
natural abilities have bad but little cultivation. Both
grades may produce equally efficient nurses, but the intel-
ectual standpoint in the two cases differs widely.
-Then as regards the lecturcB which the probationers of
to-day are privileged to hear. When can they attend them
^ith most advantage to themselves ? This is a question in-
volving the consideration of various matters relating to
Gurae8? For some inexplicable reasons it is a common insti-
tutional custom for recently admitted probationers to attend
?ctures during the first year of their training. Thus
?y way have to try and digest elementary anatomy
whilst they are still struggling with the mysteries of clinical
bermometera and striving vainly to discriminate between
receivers " and " dressing trays."
In other words, the new probationer haa the difficulties of
her ward work completely filling such of her thoughts as are
not occupied with sensations of personal weariness and the
excitement naturally attendant on the new world into which
she has entered.
At first the minds of most novices are concentrated on
their own individuality, and this is presently superseded by
the living interests around them.
From a variety of causes the first six months spent in regu-
lar training are attended with greater Belf-consciousess, more
bodily weariness, and mental excitement than any other stage
of a nursing career.
The first symptom soon disappears in a true woman, the
second is greatly reduced in degree as she getB accustomed to
the routine and seasoned to her surroundings, and the third
is gradually merged in assiduous discharge of daily duties.
It takes some women, who develop eventually into excel-
lent nurses, quite a year to "settle down" and to accept
their responsibilities unflinchingly. That poetic fiction " the
born nurse" has a foundation of truth, for assuredly some
women are sent into the world with an aptitude for tending
the sick which would make their presence welcome to
sufferers without any preliminary training. Bub when train-
ing and experience are added to this instinctive faculty we
have, indeed, a perfect nuree. Yet even such a woman as
this would enjoy good lectures and pass a far better examina-
tion during the second year spent in institution work. As
for the ordinary type of probationer, no one could hesitate in
deciding that she ought never to be required to add these to
the other strains attendant on a first year's work.
Leaving the ward weary, craving rest and fresh air, yet
knowing that neither can be enjoyed because of the near
prospect of " that dreadful exam.," is the usual condition of
things with unseasoned probationers. We have heard a
tired girl say quietly that she had to spend all her off-duty
time and even her monthly holiday in study, otherwise she
would certainly fail to pass the examination on which the
success of her future career largely depended. The nurse in
question, alas ! one of many, was an admirable worker,
anxious, conscientious, and demoted to her patients. Ten
years earlier she had left school, with a distinct feeling of
relief and never imagining that examinations would cloud
her sky again. If the ward routine were mastered first,
if the daily duties had time to become so far mechanical that
the probationer ceased to feel harassed by a single detail,
then a time would arrive when lectures would be en-
joyed and profited by, and when the inevitable examina-
tion would ceaBe to be a bugbear. The second year of a
probationer's training should therefore certainly be the time
when all lectures, save perhaps those of elementary nursing,
could, with the greatest advantage to workers and work,
take a prominent position.
flotes anb Queries.
Queries.
(141) District Nursing.?Without entering'a hospital, where can a Jady
obtain a short training in district nursing in tho North of England in
bracing climate ??Janetta.
(142) Mental.?The bo.k recommended does not give the information
I require ; please namo another.?A. E. H.
(143) General Training.?Where can I get a few months' training in
general nursicg? L.O.S.
(141) Benger.?Will some correspordent tell mo positively whether
" Banger" is pronounced with a hard or soft G P?Sister.
(145) .Brechin.?Information wanted about Indian nursing.?Anxious
Enquirer.
Answers.
(141) District Nursing. (Janetfa.)?Without previous experience
short training in district work would bo useless. We are not aware of
any association which would undertake to give snoh instruction except
to trained nurses.
(142) Mental. (A. E- H.)?Please say exactly what information you
(143) General Training, (L. 0. S)?Write to the Matrons of Royal Free
Hospital. Gray's Inn Road, and London Hospitu, Wnitechapel.
(144) Benger (Sister).?It is ordinarily pronounced with a hard G, but
as BurnameB are varied by fashion and oustom we should advise your
applying direot to the owner.
(145) Brechin (Anxious Enquirer).?You have not sent your name and
address*
xcviii THE HOSPITAL NURSING SUPPLEMENT. June 3 1893.
Sent.
Hast thou gone Badly through a dreary night,
And found no light,
No guide, no star to cheer thee through the plain,
No friend, save pain ?
Wait?and thy soul shall see when most forlorn
Rise a new morn.
?A. A Proctor.
"you go by the two o'clock train from Paddingtoc, and
change at Swindon. You have another change and an hour
to wait at Datton, so you will not be at Maraley till nearly
six."
" You do not know what the case is ? "
"No, the telegram merely says, ' Nurse wanted at once.
Philips, Marsley Green.' "
"I hope you will find the oase a pleasant one. You
must make haBte now, or you will lose the train."
Aoting on these brief instructions, I was soon on my way,
passing out of the smoke of London into the quiet English
country with its sweet green meadows and glimpses of that
river of rivers, the Thames, lying bathed in the warm glow
of an autumn afternoon.
The shadows had grown long, and the " Abendroth " was
low on the western horizon by the time the train slackened
speed and stopped at Marsley Green, a small and utterly
insignificant station, at which no other passenger alighted.
The porter made the most of the solitary traveller and
her small amount of luggage, and I fear he felt inadequately
repaid for his Budden energy by the very small tip with
which I presented him.
The one cab of the place received my boxes, and the
cabby my rather vague direction, " Philips, somewhere near
here. Do you know the name ? "
"Colonel Philips? YeB'm ; large house at the corner.
Know him? Served under him many a long year. Yes, I
know the Colonel well enough."
Satisfied I was on the right road, I fell to wondering what
my paiient would be like, and then to thinking of the case I
had left the day before, for the short stay I had made in the
house had shown me one of those chaptors of a domestic
tragedy whioh a nurse, by reason of her duties, is so often
bound to witness, and is also in honour bound to speak no word
about.
My meditations were short in the extreme. I found in a
few minutes that wo had turned into a drive which, after a
sharp curve, widened into a large gravelled space before a red-
brick house. A quiet place, no sound from within or with-
out broke the silence that seemed to wrap us round.
The door was, after a considerable delay, opened by a
small maid, who looked worried, and, I thought, rather sur-
prised to lee me.
" Colonel Philips is in ? "
"Yob, he is in. He never goes out now," she added in a
burst of confidence as if the fact was weighing very much on
her mind. "I don't think he will see anyone. He's very
queer to day."
" Oh, yes, he will see me," I answered, with great con-
fidence. " He has tent for me, and expects me this evening."
" Oh 1" replied the small domestic in a tone which implied
that after all nothing would surprise her very much. " Well,
he didn't say nothing to me. But there, he don't say much
to nobody, and I'm glad there's someone come to see after
him. It's very lonely here, and he's been very queer since
Mr. Roy was took ill."
These somewhat incoherent remarks aronBedmy curiosity
and made me anxious to see my patient without further loss
of time.
" Where shall I find Colonel Philips ? " I inquired.
The bewildered servant, who seemed more agitated than
before at the sight of my boxes, pointed- across the hall,
and then turned to pour out her grievances into the ear of
the cabman.
" Wherever them boxes is to go I don't know. Neve?
had a word of warning, and got too muoh work to do
as 'tis."
Leaving them to settle the destination of my boxes as best1
they could, I crossed the hall in the direction pointed out.
Two doors stood before me, both closaly shut. Fancying I
saw a gleam of light beneath the one to the left, I opened
it at a venture, and stepped noiselessly on to the thick
carpet.
The evening g^pw had died out of the western sky, and
the soft light that still remained was shut out of the room by
heavy curtains closely drawn.
The light that I had seen came from the fire only. The
fitful flames gleamed out of what seemed a large cavern, so
wide and deep was the old fireplace surrounded by quaint
Dutch tiles and surmounted by grotesque oak figures.
What did the dancing firelight show me ? Of what the room
contained beyond the boundary of the bright Eastern rug
which lay before the hearth, and made an oasis of warmth and
colour in the midst of the surrounding gloom, it showed me
very little, for the light could not disperse the dark shadows
which filled the corners of the Bpacious room.
And, indeed, it mattered little what objects it might have
shown, for the eye was drawn at once to the only living
creature in the room, a tall figure which stood at the side of
the hearth and leant wearily against the projecting angle of
thB old mantel-shelf.
The ' cheerful flicker danced over the sad and sunkeD
features as if to mook with their ever shifting and playful
movements the set and fixed expression on the haggard and
wasted face.
They seemed in a heartless way to make game of that;
saddest sight which can meet and startle the human eye, the
sight of a man who has lost hope, a man who carries witb
him the burden of a " life that is weary of life."
The dark leaping shadows appeared to me like mocking
imps exulting in the sight of human misery and wretchedness.
And yet that fire Bhowed the face of a man which would have
moved to deepest pity any heart which was not callous and
hardened beyond any hope of appeal to its better feeling*'
The face of a man who had struggled hard against over-
whelming odds, and who feels that the end has oome. Th?
type of who shall say how many countless numbers of
" Hearts that are broken with losses,
And weary with dragging the crosses
Too heavy for mortals to bear."
What was there, what could could there be in that man'?
life to bring that hunted look to those sunken eyes ; eye*
which " looked but did not see ;" that drawn and weary
pression to those worn and refined features ?
Whatever the mental distress may have been, physic?*
illness was doubtless there. My praotised eye noted at one?
the deep and unmistakeable traces of its cruel work. ^ft9
the restless and disturbed mind fretting through its feeble
tenement of clay, or had the pains of the body at lengtb
harassed the imprisoned aoul beyond mortal endurance -
How could I tell?
A small glass held in his hand contained a dark fluid wi^
a pungent smell.
He seemed to have forgotten that his hand held it, an(^
could have imagined, so motionless was the figure before n1?'
that the soul had for a while deserted its frail prison house*
and has sought liberty beyond the bounds of that gloomy
room.
(To be concluded.)
June 3, 1893. THE HOSPITAL NURSING SUPPLEMENT. xcix
Woe 3Book TKHorlb for IKIlomen ant> Iflurees*
[WeinTite Correspondence, Criticism, Enquiries, and Note? on Books likely to interest Women and Nurses. Address, Editor, The Hospital
(Nurses' Book World). 428, Strand, W.O.I
Kecollections of Count Leo Tolstoi. By C. A. Behrs.
(William Heinemann.)
There ia hardly a more interesting personality in Europe
than Count Tolstoi. Quite independently of his books, the
man himself has won universal sympathy by his earnest
labours among the wretched peasants in his country and by
the personal sacrifices he haB made for his creed. This
volume then of recollections which his wife's brother has
compiled will be read with curiosity and interest, perhaps
also with a little disappointment. Truth to say, the
hero of the Russian famine appears in the light of his family
Ufe in more than one particular under the ienoble aspect of
a faddist. We read, for instance, that "like Rousseau, he
held that the praotice of medicine should be made general
and not confined to one profession. Hence hiB preference
for popular medicines and for midwife remedies." This
aversion to doctors, all of whom he considered completely
ignorant of the causes or right treatment of human maladieB,
increased aB time went on. " Two years before my last
visit the Count accidentally hurt his foot. The pain became
60 intense as to make him for a while delirious. His wife then
determined to take upon herself the responsibility of sending
for a surgeon. The latter was received by his patient with scant
affability, and was roughly told, probably in the hope of getting
tid of him, that he would not have come unless he had hoped
to get a good fee. To thiB the surgeon quietly replied that he
wondered the very man who preached love to his neighbour
should himself so flagrantly violate the rule of love. In the
end the surgeon was allowed to apply his treatment, and
before long the inflammation diminished and the refractory
patient was restored to heath. But the Count remained
unshaken in his belief." Perhaps there is enough real
nobility to carry off a few unworthy prejudices of this sort.
Certainly the life of the Count, as depicted by M. Behre, is
very beautiful in its practical application to all matters small
and great of the law of love, irrespective of personal comfort
or interest. His entire system of ethicB is snmmed up in
three general principles : (1) That we should not oppose evil
with force ; (2) Tnat we should not consume more than we
ourselves produce; (3) That men and women should equally
practice and aBpire towards purity and chastity. He was
considerably hampered in the observance of the sccond rule
by the possession of a large property, and though
he refuses to receive any income or profis from it he has
been unable, owing to the protests of his wife, to prevent
his family from enjoying its benefits. They are so far, how-
ever, in accord with his principles as to devote the greater
part yearly to the relief of the poor. Bis own ideal of relief
involves the bestowal of personal labour. " I remember
how once a poor, old, decrepit monjik came and asked him to
give him some timber with which he could repair his broken-
down sheds. The Count invited me to go with him into the
forest, and we two, having taken our axes with us, cut down
some trees, lopped off the branches, and piled the logs in
THE HOSPITAL NURSING SUPPLEMENT. June 3, 1893.
THE BOOK WORLD FOE. WOMEN AND NURSES ?continued.
order on the peasant's cart. And when we had finished
and sent the moujik away rejoicing, he turned to me and
said, 'Is it possible tojdoubt the necessity of helping our
neighbour in distress, or the joy which such help brings with
it? ' " We are introduced to him in his domestic life, fetching
the water for his bath, cleaning out his own Btudy, refusing
rigidly any service from dependents, and rejecting even the
use of animals in his stern observance of the law of love.
He is of course a vegetarian, and always performs the
journey from his country house to Mobcow, a distance of a
hundred and ninety-five versts (about a hundred miles), on
foot, rather than compel the service of a horse. It is some-
thing at least to have enabled so close an observer and
intimate friend as M. Behr to assert that he "cannot
imagine how anyone, unless he be actuated by envy or malice,
will venture to deny that in every minutest point the Count
has so far as possible practised in his life what he preaches
in his boobs." How far his teaching conduces to the welfare
of his disciples is a question which the future only can decide.
It is something that the teacher is sincere.
Through Connemara in a Governess Cart. By the
Authors of " An Irish Cousin." (W. H. Allen and Co.)
This lively volume gives an account of the adventures of
two Irish ladies, who disgusted with their experience of a
wet June in London, resolved suddenly to " get an ass and
an ass-car, or Bome sort of a yoke whatever and drive
through Connemara.'' The aventures are of no very Btart-
iDg nature, but the writers have the knack of making each
day's doings amusing, however unimportant. They have
avoided the common error in books of this description of
making too much out of small incidents, and write always
lightly and without effort. It is only to be regretted that
the easiness of the style degenerates sometimes into flippancy.
There is a good deal of humour in the presentment of libble
characteristic traits and spirited bits of dialogue, a humour
which finds expression also in the clever sketches with
which the book is sprinkled. The initial difficulties
of finding a suitable conveyance at Oughterard for the
expedition being at length surmounted, the two ladies set out
in the governess's cart, drawn by a long-legged, long-earned
animal, described by the owner as " a little giddy about the
head, but if ye'll not touch the ears she's the quietest little
thing at all." This rather ominous commendation prepares
the reader for future complications. But Sibbie, after
various escapades, turns fat and lazy under large diet and
tender treatment to the point of provoking from an onlooker
towards the close of the tour the remonstrance, " Ah, don'fc
be sparin' him that way, ladies. He's well able to pull the
pair of ye ; nourish him with the whip." The exact route of
the travellers it is not always easy to trace, nor does it matter
in the least. They work thair way back by degrees to Ough-
terard, the point from which they started, " the best village
for its size this side of Galway," say s the second cousin. " And
the place has improved so wonderfully. For instance, there's
the widows' almshouse. It isn't so very long ago since an
old woman said to my grandmother, 'That's the widdies'
almshouse, and sorra widdy in it but one little owld man,'
and now it's simply bursting with widows."

				

